# A man with an infected finger: a case report

**DOI:** 10.1186/s13256-015-0589-5

**Published:** 2015-05-23

**Authors:** Pieter J Gathier, Titus JA Schönberger

**Affiliations:** 1 Jeroen Bosch Ziekenhuis ’s-Hertogenbosch, Henri Dunantstraat 1, 5223 GZ ’s-Hertogenbosch, The Netherlands; 2 Beatrixziekenhuis Gorinchem, Gorinchem, The Netherlands

**Keywords:** Dermatology, Emergency medicine, Herpetic whitlow, Surgery, Whitlow

## Abstract

**Introduction:**

Whitlow is an infection of a finger or around the fingernails, generally caused by bacterium. However, in rare cases, it may also be caused by the herpes simplex virus. As herpetic whitlow is not seen often, it may go under-recognised or be mistaken for a different kind of infection of the finger. Delayed recognition and/or treatment puts patients at risk of complications ranging from superinfection to herpetic encephalitis.

**Case presentation:**

A 23-year-old Caucasian man with no medical history was referred by his primary care physician because of erythema and swelling of the little finger of his left hand. The primary care physician had already treated him with the oral antibiotic Augmentin® (amoxicillin-clavulanic acid) and incision of the finger, but this had not resolved his complaints. He had multiple vesicles on the finger, which led to the diagnosis of herpetic whitlow, which we confirmed by polymerase chain reaction testing. All cutaneous abnormalities disappeared after treatment.

**Conclusions:**

Whitlow is rarely caused by the herpes simplex virus, but this disease requires a swift recognition and treatment to prevent complications. This case serves to emphasise that not all whitlow is caused by a bacterial infection, and that it is important to differentiate between herpetic and bacterial whitlow, as these diseases require a different treatment.

## Introduction

Herpetic whitlow is an infrequently seen cause of infection of a finger. It is estimated to affect 2.5 per 100,000 people each year. It is caused by the herpes simplex virus type 1 (60%) or type 2 (40%). When it is seen, it is generally in patients with occupational hazards such as medical workers, dental workers, hairdressers or thumb-sucking children. As herpetic whitlow is rare, it may go under-recognised or be mistaken for a different kind of infection of the finger. Delayed recognition and/or treatment puts patients at risk of complications ranging from superinfection to herpetic encephalitis [1,2]. In patients with specific occupational hazards or those with an atypical whitlow, physicians should be aware of herpetic whitlow.

## Case presentation

**Figure 1:**
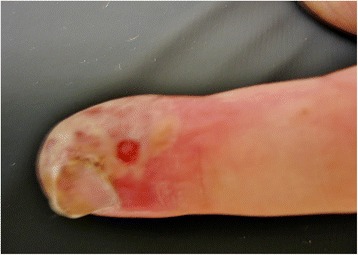
Patient's finger, dorsal view. Dorsal and radial side of the patient's little finger, showing yellowish vesicles and erythema.

**Figure 2:**
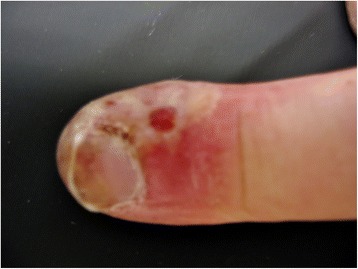
Patient's finger, dorsal view (Figure 1) zoomed in.

A 23-year-old Caucasian man with no medical history was referred to our emergency department by his primary care physician because of swelling and erythema of the little finger of his left hand for ten days prior to presentation. He had been prescribed the antibiotic Augmentin® (amoxicillin-clavulanic acid), and the primary care physician had already incised the finger releasing clear liquid (no pus). Despite this therapy, his complaints persisted. He had no fever or itching, and had never had these complaints before (Figures 1 and 2).

**Figure 3:**
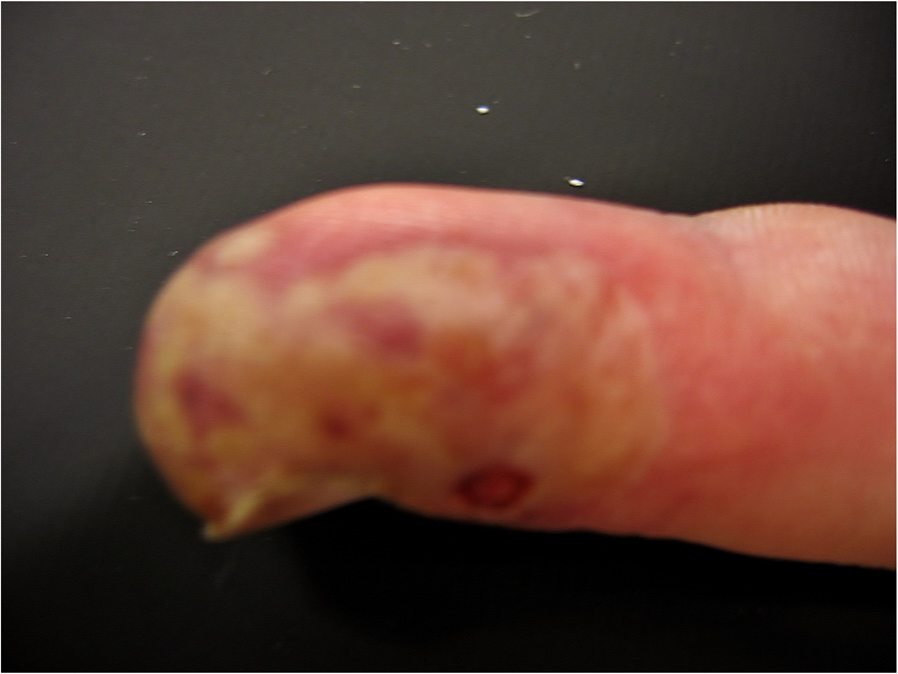
Patient's finger, radial view. Radial side of the patient's little finger, showing yellowish vesicles and erythema.

**Figure 4:**
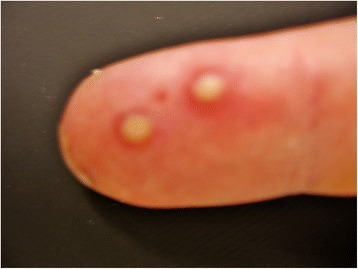
Patient’s finger, palmar view. Palmar side of the patient's little finger, showing two yellowish vesicles.

We confirmed the erythema and swelling of the distal phalanx of his left little finger, with vesicles with a yellow translucent colour. From these vesicles, a clear fluid spontaneously discharged. There was no pus, bony tenderness or pain over his flexor tendons. The motion of his finger was unlimited, and he had no fever (Figures 3 and 4).

On the basis of the clinical appearance we considered herpetic whitlow with superinfection. When asked, he confirmed he had also observed vesicles on his genitals. He denied ever having sexual intercourse or contact with infected individuals. A polymerase chain reaction on herpes simplex virus type 1 was positive on both the material from his genital vesicles and on material from his finger. We referred him to a dermatologist for further treatment. Because of spontaneous subsidence of his complaints, Fucidine® (fusidic acid) cream was chosen as primary treatment. In other cases, in which subsidence does not occur spontaneously, antiviral agents such as acyclovir or valacyclovir may also be used.

The herpes simplex virus causes two types of infections: primary and recurrent. Usually, a break in the skin barrier (for example a wound) allows the virus to enter the tissue and establish an infection. Appearing several days after a person's first exposure, the sores of a primary infection last approximately 1 to 3 weeks. They heal completely, rarely leaving scars. Nevertheless, after the primary infection, the virus remains in the body, hibernating in nerve cells. Certain triggers can cause the hibernating (latent) virus to become active and travel back to the skin. Recurrent infections tend to be milder than primary infections, and generally occur in the same location as the primary infection [3,4].

## Conclusions

Whitlow is rarely caused by the herpes simplex virus, but this disease requires swift recognition and treatment to prevent complications. As this treatment differs from that of a traditional whitlow, physicians should be aware of herpetic whitlow.

## Consent

Written informed consent was obtained from the patient for publication of this case report and any accompanying images. A copy of the written consent is available for review by the Editor-in-Chief of this journal.
